# Bright spots for advancing ecological understanding and conservation decision‐making

**DOI:** 10.1111/cobi.70109

**Published:** 2025-07-23

**Authors:** Holly S. Embke, Zachary S. Feiner, Gretchen J. A. Hansen, Daniel Isermann, Olaf P. Jensen, Christopher I. Rounds, Quinnlan C. Smith, M. Jake Vander Zanden

**Affiliations:** ^1^ Midwest Climate Adaptation Science Center U.S. Geological Survey St. Paul Minnesota USA; ^2^ Office of Applied Science Wisconsin Department of Natural Resources, Science Operations Center Madison Wisconsin USA; ^3^ Center for Limnology University of Wisconsin–Madison Madison Wisconsin USA; ^4^ Department of Fisheries, Wildlife, and Conservation Biology University of Minnesota St. Paul Minnesota USA; ^5^ U.S. Geological Survey, Wisconsin Cooperative Fishery Research Unit, College of Natural Resources University of Wisconsin–Stevens Point Stevens Point Wisconsin USA

**Keywords:** adaptation, hotspots, natural resources, social–ecological systems, adaptación, puntos calientes, recursos naturales, sistemas socio‐ecológicos, 适应性, 社会-生态系统, 热点区域, 自然资源

## Abstract

A lot can be learned by studying bright spots—defined as unexpected positive outcomes. In fields like public health, education, and oncology, identifying factors behind bright spots reveals previously unknown drivers of success that can be replicated elsewhere. This concept is being applied in conservation but is hampered by variations in definitions of *bright spots* and confusion with hotspots—sites with high absolute values of a metric. We developed a framework to clearly define and distinguish between hotspots (e.g., a wetland with high plant diversity) and bright spots (e.g., a biodiverse wetland in a housing development), which outperform conservation expectations. The framework is an iterative cycle, consisting of setting expectations for relative comparisons, classifying systems into bright, dark, hot, and cold categories, and digging deeper to reveal hidden mechanisms and opportunities for intervention. We drew on examples from diverse fields to demonstrate how our framework can generate new knowledge, identify potential interventions, and inform management priorities. Defining conservation and management expectations, often through predictive models, is essential to understanding drivers of success and fosters hypotheses about overlooked factors. Our framework can enhance ecological understanding, guide interventions, and help prioritize actions in conservation and natural resource management.

## Abstract (es)

Hechos positivos para el progreso del entendimiento ecológico y las decisiones de conservación

**Resumen:**

Podemos aprender mucho si estudiamos los hechos positivos, es decir, los resultados efectivos inesperados. En campos como la salud pública, la educación y la oncología, la identificación de los factores que explican los hechos positivos revela factores de éxito desconocidos hasta ahora que pueden reproducirse en otros campos. Este concepto se está aplicando en la conservación, pero se ve obstaculizado por las variaciones en las definiciones de los hechos positivos y la confusión con los puntos calientes, es decir, los lugares con altos valores absolutos de una métrica. Desarrollamos un marco para definir y distinguir claramente entre puntos calientes (por ejemplo, un humedal con una gran diversidad de plantas) y hechos positivos (por ejemplo, un humedal biodiverso en una urbanización), que superan las expectativas de conservación. El marco es un ciclo iterativo que consiste en establecer expectativas para comparaciones relativas, clasificar los sistemas en categorías brillantes, oscuras, cálidas y frías, y profundizar para revelar mecanismos ocultos y oportunidades de intervención. Con base en ejemplos de diversos campos para demostrar cómo nuestro marco puede generar nuevos conocimientos, identificamos posibles intervenciones y fundamentamos las prioridades de gestión. La definición de las expectativas de conservación y gestión, a menudo mediante modelos predictivos, es esencial para comprender los factores que impulsan el éxito y fomenta la formulación de hipótesis sobre factores pasados por alto. Nuestro marco puede mejorar la comprensión ecológica, orientar las intervenciones y ayudar a priorizar las acciones de conservación y gestión de los recursos naturales.

## 促进生态认知与保护决策的亮点

**【摘要】** 通过研究亮点(即意外的积极成果)可以获得许多启示。在公共卫生、教育和肿瘤学等领域, 识别亮点背后的因素可以揭示此前未发现且可能在其他地方复刻的成功驱动因素。这一概念正被应用于保护领域, 但仍受限于“亮点”定义的差别以及与热点(即某一指标绝对值较高的地点)的混淆。本研究开发了一个框架, 明确界定并区分了热点(如植物多样性高的湿地)与亮点(如住宅区内生物多样性高的湿地), 其中后者有更高的保护预期。我们的框架是一个迭代循环, 包括设定相对比较的预期、将系统划分为亮点、暗点、热点和冷点类别, 以及深入挖掘以揭示隐藏机制和干预机会。我们通过不同领域的案例展示了该框架如何产生新知识、识别潜在干预措施, 并指导管理优先保护。通过预测模型等方法明确保护与管理预期, 对于理解成功驱动因素并提出关于被忽视因素的假设至关重要。我们的框架可加强生态认知、指导干预措施, 并帮助确定保护与自然资源管理中优先行动。**【翻译:胡怡思;审校:聂永刚】**

## INTRODUCTION

Outliers can offer valuable insights. In their book *Made to Stick*, Heath and Heath (2008) argue that during times of change, focusing on “bright spots” (i.e., unpredicted areas of success) can lead to important discoveries. They ask, “What contributes to these bright spots, and can we replicate them?” (Heath & Heath, 2007; Pascale et al., 2010). This approach originated in public health (e.g., Zeitlin, 1991) and was designed to uncover solutions to seemingly unsolvable problems (e.g., Pascale et al., 2010). It has since been applied in philosophy (e.g., Macy & Gahbler, 2006) and medicine (e.g., Banerjee et al., 2022) and has gained popularity for use in understanding exceptional people (e.g., Buettner, 2009; Gladwell, 2011; Heath & Heath, 2007) (Table 1). This emphasis on areas of success can inspire proactive change across sectors (Bennett et al., 2016; Cvitanovic & Hobday, 2018; Jeanson et al., 2021).

**TABLE 1:** Examples of bright spot terminology used across disciplines.

Discipline	Source	Term used	Term definition	Application	Approach used
Agriculture	Burchfield & Schumacher, 2020	Bright spots	Counties exceeding production expectations	Understand adaptive practices for food security	Quantitative analysis
Agriculture	Frei et al., 2018	Bright spots	Agricultural areas exceeding expectations for biodiversity and landscape multifunctionality	Evaluate spatial overlap between bright spots of ecosystem services and biodiversity	Quantitative analysis
Communication and psychology	Gladwell, 2011	Outlier	Exceptionally successful people	Examine the factors that contribute to high levels of individual success	Case study analysis
Communication and psychology	Heath & Heath 2008	Sticky idea	An idea that is understood, is remembered, and has a lasting impact on the audience's behavior	Understand why some ideas resonate and explain ways to make ideas stickier	Case study analysis
Conservation—amphibian	Scheele et al., 2019	–	–	Identify priority conservation areas for amphibians based on disease resilience	Quantitative analysis
Conservation—freshwater	Garrah et al., 2019	Bright spots	Lakes exceeding expected ecological condition	Support watershed management prioritization	Quantitative analysis
Conservation—general	Bennett et al., 2016	Bright spots	Initiatives or places inspiring hope for a better future	Frame positive visions for sustainability pathways	Visioning and community engagement
Conservation—general	Post & Geldmann, 2018	Exceptional responders	Conservation projects that succeed beyond expectations	Learn from high‐impact conservation efforts to guide future efforts	Case study analysis
Conservation—marine	Gilby et al., 2023	Bright spots	Coastal sites with superior ecological conditions	Guide conservation prioritization	Quantitative analysis
Conservation—marine	Lester et al., 2020	Bright spots	Coral reefs showing better‐than‐expected ecological performance	Highlight potential for coral reef recovery or protection	Quantitative analysis
Conservation—marine	O'Leary et al., 2017	Bright spots	Resilient coastal ecosystems despite climate change	Understand climate change resilience of coastal marine ecosystems to guide conservation actions	Quantitative analysis
Fisheries	Cinner et al., 2016	Bright spots	Coral reef fisheries performing better than expected under pressure	Identify resilience factors for fisheries management scaling	Quantitative analysis
Fisheries	Jeanson et al., 2021	Bright spots	Recreational fisheries inspiring hope for a better future	Identify innovative adaptation approaches in recreational fisheries in the face of climate change	Case study analysis
Fisheries	Kovalenko et al., 2019	Bright spots	Sites with unusually high functional and taxonomic diversity of fishes	Identify habitat features promoting diversity	Quantitative analysis
Fisheries	Schiller et al., 2024	Positive deviants	Fisheries with better outcomes despite similar constraints	Identify enabling practices or governance models to support fisheries sustainability	Case study analysis
Fisheries and limnology	Hansen et al., 2022	Resilient locations	Lakes expected to maintain coldwater habitat under change	Conservation targeting under climate change	Quantitative analysis
Health and wellness	Buettner 2009	Blue zones	Places where people live significantly longer, healthier lives	Understand lifestyle and environmental contributors to longevity	Case study and demographic analysis
Medicine	Banerjee et al., 2022	Bright spots	Geographic areas outperforming others in health outcomes despite challenges	Identify factors improving diabetes management	Qualitative analysis
Philosophy	Macy & Gahbler, 2010	Stories of change	People who have brought about transformative changes	Individual stories of resilience to promote hope and agency in social and environmental movements	Case study analysis
Policy	Cvitanovic & Hobday, 2018	Bright spots	Positive cases at science–policy–practice interface	Build optimism and improve science uptake	Qualitative analysis
Public health	Zeitlin, 1991	Positive deviance	Children who demonstrate above‐average growth in impoverished environments	Understand why some children show greater growth despite impoverished conditions	Community engagement
Public health and organizational behavior	Pascale et al., 2010	Positive deviance	The few individuals in a group who find unique ways to look at, and overcome, seemingly insoluble difficulties	Discusses problem‐solving approach based on learning from individuals who overcome great challenges	Case study and demographic analysis
Urban planning	Wolch et al., 2014	–	–	Address equity and justice in urban green‐pace planning	Case study analysis

*Note*: The review of terms is not comprehensive; rather, it is a starting point for exploring cross‐disciplinary interpretations and applications of the concept that formed the foundation for the bright spots framework.

The bright spots approach is being increasingly applied in conservation and natural resource management, though often under differing understandings (Table 1). For instance, Schiller et al. (2024) identify factors associated with “positive deviants” in global fisheries, Post and Geldmann (2018) recommend identifying “exceptional responders” for conservation, and Cinner et al. (2016) identify coral reef bright spots that are outperforming expectations. *Bright spot* is often conflated with *hotspot* (i.e., areas of high values of a metric), which is commonly used in conservation to define areas with high levels of biodiversity (Myers et al., 2000). However, hotspots differ from bright spots in that they do not account for performance relative to expectations. As the application of the bright spots concept grows and its potential to solve complex problems becomes evident, there is a need for conceptual clarity and a framework to guide the identification and application of bright spots in conservation.

**FIGURE 1:**
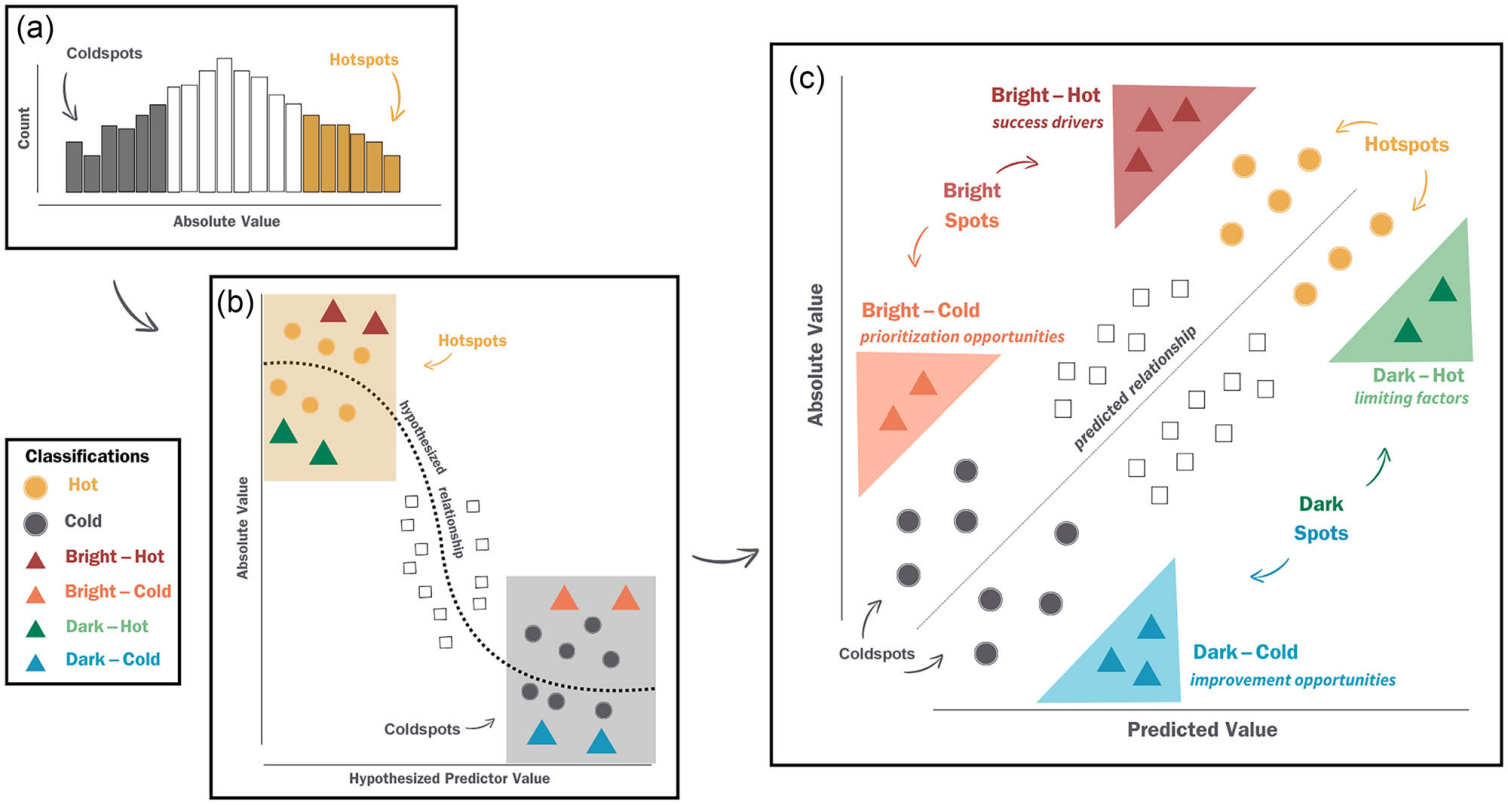
Bright spots (i.e., unexpected positive outcomes) framework founded on the relationship between absolute values of a response metric relative to predicted values of a response metric: (a) frequency distribution of absolute values of a metric, (b) absolute values from the frequency distribution in (a) when modeled with a hypothesized predictor metric that can be used to identify bright and dark spots (i.e., unexpected negative outcomes) along a hypothesized predicted relationship, and (c) relationship in (b) used to inform quantitative classifications of bright and dark spots (triangles) and hot‐ and coldspots (circles) (combined classification [e.g., dark–cold], significant deviations from the predicted relationship; squares, values of a response metric that are neither bright and dark nor hot and cold because they fall along the predicted relationship and are not extreme in absolute or predicted values; italics, examples of management relevant insights). Concepts are illustrated primarily with a linear relationship for simplicity. The relationship between predictor and response variables can take many shapes, which can influence how spots are identified and interpreted.

**FIGURE 2:**
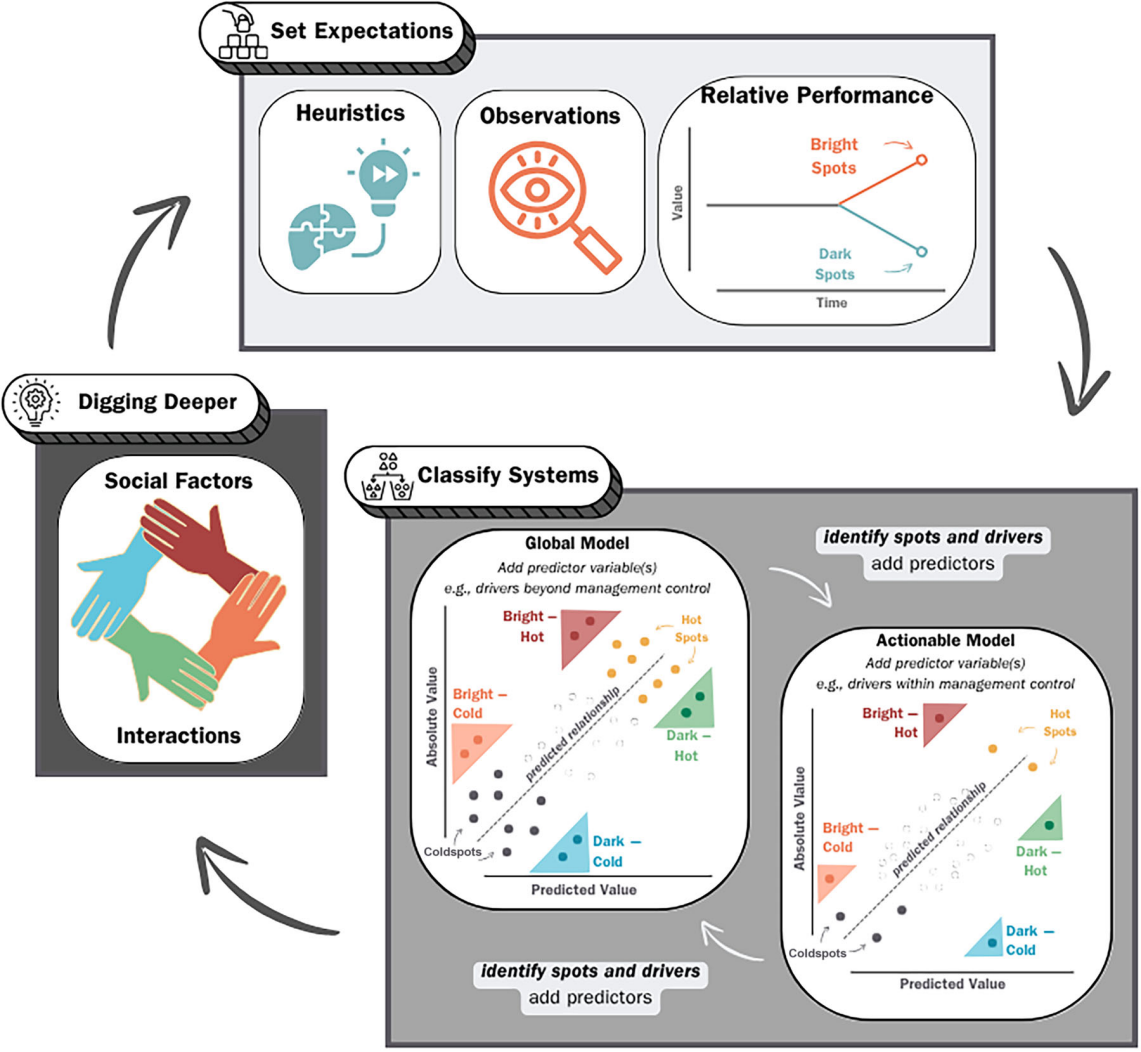
Bright spots framework for identification of bright (i.e., unexpected positive outcome) and dark (unexpected negative outcome) spots of conservation and of potential intervention opportunities through an iterative process.

**FIGURE 3:**
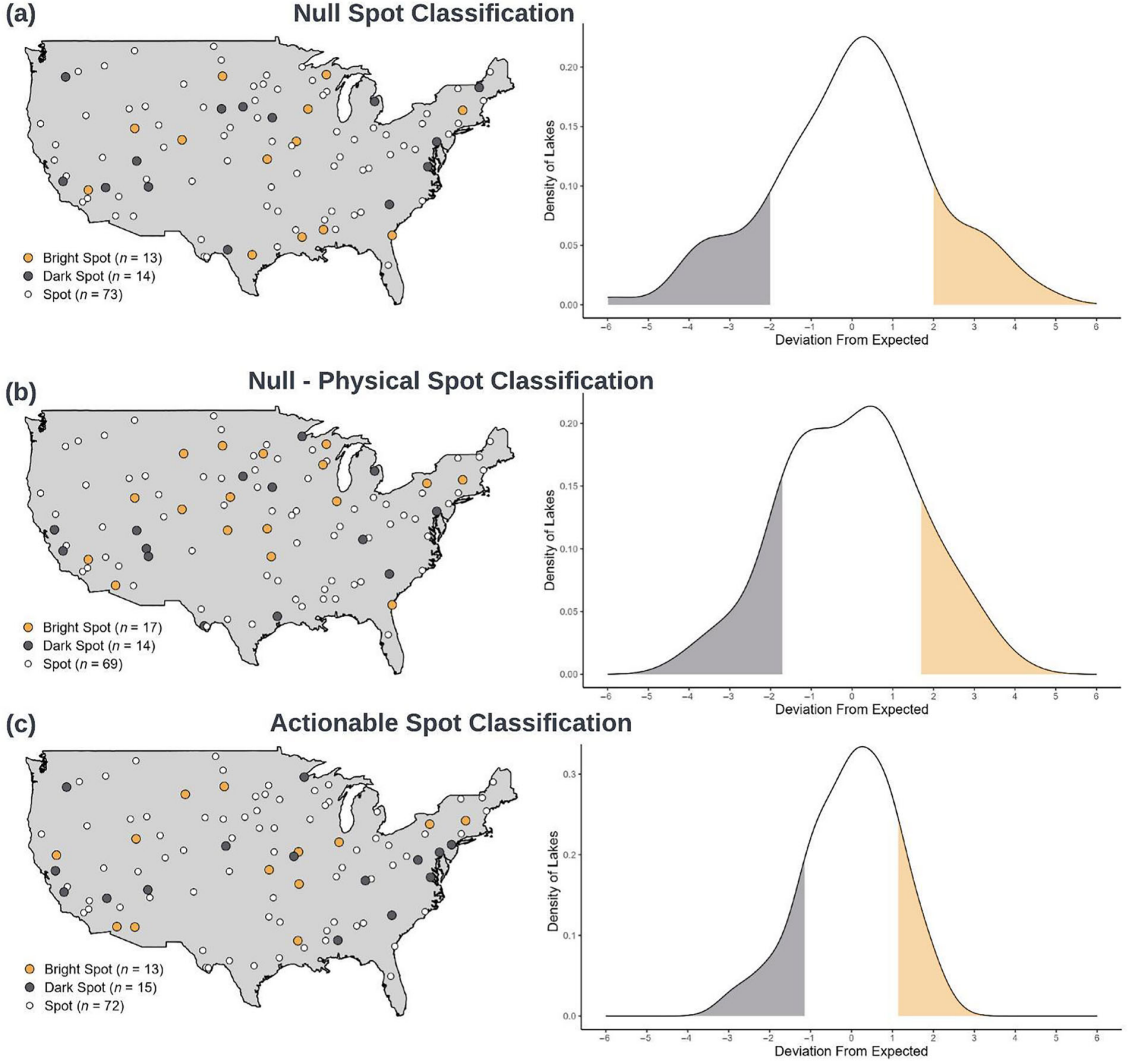
An example application of the framework for iteratively classifying conservation bright (i.e., unpredicted positive outcomes) and dark (i.e., unpredicted negative outcomes) spots to lake water clarity in the United States: (a) null model (no predictor variables), (b) a null physical model (nonactionable predictor variable, e.g., a factor beyond local control, here lake size), and (c) actionable model (factor within local control, here land use index corresponding to percentage of developed shoreline) (spot, i.e., predictable outcome). The tails and spread of deviation from expectation get smaller as models move from null to actionable (i.e., additional predictor variables are included).

**FIGURE 4:**
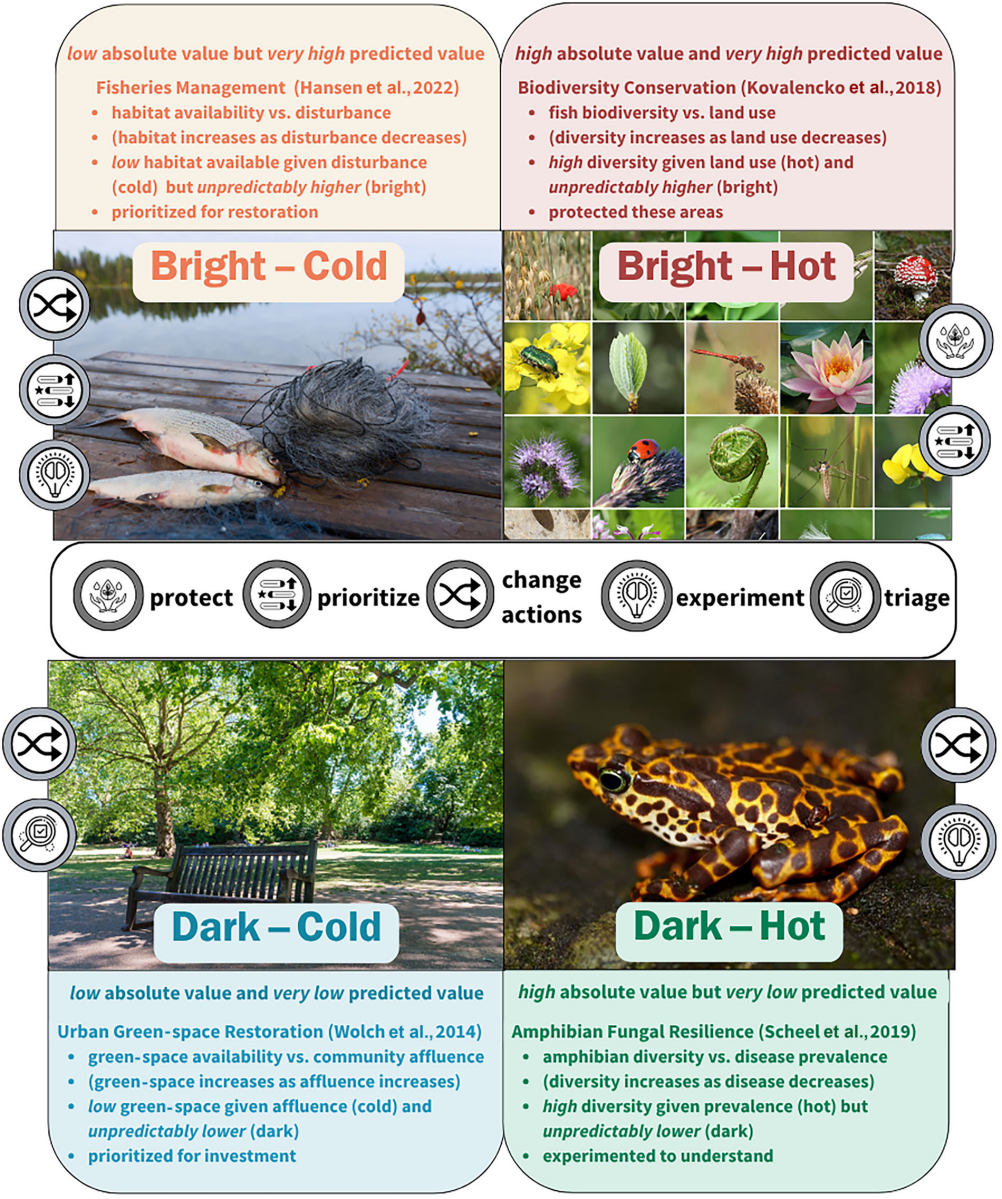
Classification of bright (i.e., unpredicted positive outcome), hot (i.e., high absolute value of metric), dark (i.e., unpredicted negative outcome), and cold (i.e., low absolute value of metric) spot classifications and examples of their use in conservation and natural resource management (example topic and citation first line above bulleted lists; bulleted lists, response metric vs. predictor metric; expectations, in parentheses; classification example, practitioner decision; actions, legend in middle of figure). All photographs are public domain.

We developed a conceptual framework for bright spots in conservation and natural resource management. We devised unified definitions of *bright spots*, *dark spots*, *hotspots*, and *coldspots* (Figure 1; Table 2) and a transdisciplinary approach to identify each type and explore their implications for conservation practitioners (Figures 2 & 3). Drawing from diverse fields, including fisheries management, biodiversity protection, urban green‐space restoration, and amphibian conservation, we sought to demonstrate how our framework serves as a powerful tool for generating new insights and ecological knowledge from outliers (Table 1; Figure 4).

**TABLE 2:** Glossary of terms used in the bright spots framework.

Term	Definition
Bright spot	System with unpredictably high absolute values of a response metric (i.e., positive outlier); inverse of a dark spot
Coldspot	System with low absolute values of a metric with no reference to model expectations; inverse of a hotspot
Dark spot	System with unpredictably low absolute values of a response metric (i.e., a negative outlier); inverse of a bright spot
Hotspot	System with high absolute values of a metric; inverse of a coldspot; in contrast with bright spots, hotspots do not consider deviations from expectations; in the absence of expectations, a hotspot is the same as a bright spot
Response	Variable of interest (dependent variable) being subject to a bright spot analysis; many possible response metrics (e.g., for an inland fishery, response metrics could include catch rate, size structure, presence of natural reproduction, or extent of recreational use)
Spot	Unit of analysis; can be an individual or set of systems, populations, or locations
System (see also spot)	Unit of analysis or observation in an analysis

## DEFINING BRIGHT SPOTS, HOTSPOTS, DARK SPOTS, AND COLDSPOTS

We made our conceptual framework for identifying systems as bright spots, hotspots, dark spots, and coldspots as cross‐site and comparative in nature (i.e., we considered multiple sites and systems of interest). These sites or systems could be fish stocks, forest patches, or wetlands. For this framework, we also identified a metric of interest. The metric is something people care about and manage for; examples include fish abundance, plant species richness, and water clarity. Broadly, we sought to assess and compare values of the metric among sites and to advance fundamental understanding of the drivers of the chosen metric to manage and conserve it.

From a simple frequency histogram of the metric of interest (Figure 1a), one can identify hotspots as sites with high values of a metric and coldspots as sites with low values of the metric. In conservation planning, resources are likely focused on conserving biodiversity hotspots because of their high conservation value (Myers et al., 2000). However, new understanding of underlying factors that influence the response metric may be missed if hot‐ and coldspots are not identified. For example, determining that habitat fragmentation is related to low animal abundances (coldspot) may indicate potential intervention options, such as reconnecting migratory pathways, but it does not provide understanding of locations that are relatively resilient to this general relationship (i.e., spots that are bright and cold [hereafter bright–cold]).

We attempted to explain variability in the metric of interest by linking it to other variables that we hypothesize influence its value. In this step, the metric of interest becomes a response or dependent variable as a function of some other metric. For a bird species richness metric, we envisioned a statistical model in which disturbance to forest patches is negatively related to bird species richness (Figure 1b). In this statistical model, some variability in species richness is explained by forest disturbance, and several sites have high residual values, both positive and negative. The simple example in Figure 1b can be expanded to include multiple predictor variables and any shape of relationship, and we generated a plot of predicted versus observed values of the response metric (Figure 1c). This plot provides the basis for classifying a site as a bright spot or a dark spot. A bright spot is any positive outlier—in other words, a site performing considerably better than predicted. A dark spot is a negative outlier, that is, a system performing considerably worse than predicted (Figure 1c).

Because high values of the response metric are hotspots and low values are coldspots, we then identified different combinations of bright–dark spots and hot–cold spots. Sites that are high performing (e.g., high absolute values) and represent positive outliers (e.g., unpredictably high predicted values) are bright–hot spots (Figure 1c, purple). Alternatively, sites may be low performing (e.g., low absolute values) but still be positive outliers. These are bright–cold spots (Figure 1c, orange). Sites that are high performing but negative outliers are dark–hot spots (Figure 1c, green). Sites that are low performing and negative outliers are dark–cold spots (Figure 1c, blue).

In this framework, a site can be classified in terms of combinations of bright, dark, hot, and cold. The highest absolute values of the response metric are hotspots, whereas the lowest absolute values are coldspots. Many sites in the middle would be classified as neither hot nor cold. Similarly, with bright–dark spots, many sites will fall near the line in Figure 1 and will not be outliers, and thus are neither bright nor dark spots.

## IDENTIFYING AND QUANTIFYING SPOTS

Our bright spots framework can be used in an iterative cycle composed of 3 steps: first, set expectations to lay the foundation for relative comparisons; second, classify systems into bright, dark, hot, and cold categories; and third, dig deeper into factors contributing to bright and dark classifications to reveal hidden mechanisms and opportunities for intervention (Figure 2). This process may continue in an adaptive interpretation loop by including additional information to clarify expectations, update models, and gain new insights into factors contributing to unpredicted results to inform conservation decision‐making.

### Setting expectations

All bright and dark spot classifications are relative to expectations; therefore, setting expectations is the first step prior to classification and understanding. Expectations may be set in a variety of ways, such as past performance, heuristics, observations, or predicted outcomes (see “Classifying systems” and Figure 2). To operationalize bright and dark spots in an actionable framework, it is beneficial to develop methods that clearly quantify which sites are bright or dark. Bright and dark spots are exceptional (good or bad) relative to their predicted response, but what that predicted response is and how it is determined vary depending on the analysis approach. For example, if the expectation is relative to past performance, bright spots may be places that are performing unpredictably better than they were previously.

Clearly defining the expectations on which bright and dark classifications are based sets the foundation for a deeper understanding of mechanisms and potential intervention. In most applications, these larger values of a response metric are classified based on their exceptional nature, implying that values must be much greater than average to be considered. The threshold separating these outliers can be context specific and set based on previously agreed‐on criteria (e.g., residuals ≥2 SD from the predicted mean [Cinner et al., 2016 or Lester et al., 2020]). Alternatively, residuals that exceed some percentage of the predicted value of the response metric (e.g., at least 50% greater than predicted) may be used, although the development of these criteria will be case specific and should consider the strengths and weaknesses of different approaches. Visual inspection of residual distributions for step changes or extreme values may also be used when qualitative assessment is sufficient (e.g., Schiller et al., 2024). The choice between these approaches depends on, for example, the nature of the data and the purpose of the analysis. The SD = 2 threshold may be most useful for large datasets with approximately normal distributions and a need for objectivity; residual‐based definitions may be appropriate when a strong theoretical or empirical expectation exists and the goal is to identify over‐ or underperformance; and visual inspection may be more appropriate in exploratory contexts or when nonlinearity and local knowledge are important. Overall, deviance from expectation is a continuum, and where one draws the line in defining a spot as bright or dark is context dependent. Bright–dark spot identification can also incorporate more complex statistical methods depending on data availability and context.

### Classifying systems

The framework generates expectations for a metric (e.g., biodiversity in national parks), and the difference between predicted and observed values of the response metric determines whether a system is a bright spot (better than predicted), a dark spot (worse than predicted), or neither (performing as predicted). Classifications can initiate an iterative modeling process where the model can be updated at any point to understand factors influencing spot classifications (Figures 2 & 3). Classifying bright spots can reveal limitations of a model (i.e., knowledge of a system and its drivers) and identify characteristics leading to deviations from expectations.

### Bright spots for action

In this iterative process, additional variables can be incorporated into a predictive model, potentially changing the classification of spots. As understanding of how ecosystems work and models improve, unexplained variance is expected to decrease. Thus, expectations may change, and what was once a bright spot may become a system that performs as predicted. If a bright spot is no longer bright after adding new predictors, this suggests that these factors are important components of success. Examining which bright spots cease to be bright with new predictors allows one to develop management strategies likely to promote high performance. Similarly, dark spots may teach one about critical factors limiting success. One knows a missing factor has been found when its inclusion in the model causes dark spots to disappear. Conversely, persistent bright spots suggest unknown drivers of success, requiring an examination of additional factors (see “Digging deeper”). Theoretically, if one's understanding of which factors regulate performance is perfect, bright and dark spots may cease to exist altogether (depending on how spots are designated [Figure 3]). In thinking of the bright spots framework as a dynamic process, there are no bright or dark spots, just spots one does not yet understand.

We used our iterative process to identify bright and dark spots across a landscape. With the inclusion of additional predictor variables, the classification (and therefore location) of bright and dark spots changes. To demonstrate this process, we used simulated water clarity (i.e., Secchi depth) data for lakes across the United States and potential predictor variables (i.e., waterbody size, land use index).

First, we developed a null model consisting of no predictor variables, where the distribution of water clarity data corresponded to bright and dark spot classifications (e.g., 1 SD from the mean is a bright spot [Figure 3a]). Second, we developed a model that included a nonactionable predictor variable (e.g., a factor beyond local control, here waterbody size [hectares]) to identify water clarity bright and dark spots (Figure 3b). Distinct from the null model approach (Figure 3a), bright and dark spot classifications were relative to the predicted relationship between current and predicted performance (e.g., 1 SD from the predicted relationship between Secchi depth and waterbody size is a bright spot [Figure 3b]). Third, we developed a model including an actionable predictor variable (e.g., a factor within local control, here land use index corresponding to percentage of developed shoreline [Figure 3c]). Similar to the nonactionable model used in Figure 3b, spot classifications were relative to the predicted relationship between current and predicted performance. We used linear regressions for illustrative purposes in Figure 3b,c. Throughout this iterative classification process, the number of bright and dark spots remained the same because of their definition based on SD; however, locations shifted spatially. Both the raw residuals and the residual SD got smaller, which resulted in approximately the same number of spot classifications in each category. The variability in classification location and type depended on model structure and predictor variables, highlighting the relative importance of nonactionable and actionable factors in informing bright and dark classifications. Although the illustration with a linear regression and only 2 potential predictor variables is simple, the iterative nature of the approach is flexible depending on context‐specific modeling approaches and data availability.

Managed ecosystems are often influenced by factors beyond local control (e.g., climate change, hydrology). However, local action may alter the trajectories of these systems and maintain key ecosystem services (e.g., Carpenter et al., 2017; Scheffer et al., 2015), and the bright spots framework could be used to identify potential management levers. For example, the first iteration of a classification of bright spots could include only nonactionable variables to estimate the predicted value of a response metric. Once these nonactionable factors have been incorporated, additional actionable variables may be added to the model to determine which variables alter predicted responses and used to identify management levers. For instance, a model of lake water quality could include nonactionable factors of lake size and actionable measures of watershed land use to identify locations in which watershed protection or restoration is likely to affect water quality, given the biophysical context of the systems of interest (Figure 3). This method, combining biophysical modeling and actionable characteristics analysis, has identified characteristics of successful lake associations (Garrah et al., 2019) and agricultural production (Burchfield & Schumacher, 2020; Frei et al., 2018). In cases where no actionable factors are identified, the next best step may be to explore hidden contextual drivers or unmeasured variables that could explain system performance and guide future inquiry (see “Digging deeper”) or accept the system in its given state.

Although the simplest bright spots application may focus on exceeding expectations based on a single response metric, the concept has also been applied using multiple response metrics (Gilby et al., 2023). For example, Frei et al. (2018) evaluated spatial overlap in 2 response metrics: bright spots of ecosystem services and avian biodiversity. They found that places that were bright spots for one response metric were often bright spots for the other (a hotspot of bright spots) (Frei et al., 2018). Using multiple response metrics to identify bright spots, especially across biotic, abiotic, and social dimensions, may provide insights into ecological mechanisms as well as potential management strategies.

We treated bright spots as static classifications or as classifications that can change with updated models. However, ecosystems are dynamic and the bright spots framework can be as well. Today's dark spots may become tomorrow's bright spots and vice versa, either through management intervention or social–environmental change. For instance, some agricultural fields may host high migratory bird diversity during the nonbreeding season but not in the breeding season, making them bright spots at one time of year and dark spots at another. Information may be gained by tracking how the relative brightness of spots changes over time or during disturbance. For example, it may be useful to conceptualize bright spots of resilience as conservation units that defy expected degradation over time or rebound quickly from a disturbance. O'Leary et al. (2017) applied this concept to climate change resilience of coastal marine ecosystems, defining bright spots as areas where habitat was maintained after climatic disturbance. Here, resilience was assumed to be rare (e.g., the expectation used to identify bright spots), so systems recovering after disturbance were deemed bright spots. With available data, models can be refined to focus on expectations of trend or resilience rather than of status alone.

Many previous bright spots approaches do not consider temporal variability, though some have incorporated time by averaging repeated (e.g., annual) observations for a given site (Garrah et al., 2019). However, this approach loses potentially valuable information on within‐site variability. Further, forecasting bright spots is limited by the need to compare observations to future expectations. Exploring statistical methods that incorporate spatial and temporal variability into bright spots classification may be key to understanding system status and potential management interventions. Classifying systems to determine outliers (bright and dark spots) is only the beginning to understand underlying mechanisms and potential interventions.

### Digging deeper

Following classification of bright and dark spots, one can dig into the factors contributing to unpredicted outcomes. Bright spot analyses are grounded in the premise that there are unknown drivers that cannot be easily seen or understood by simply classifying systems (i.e., there is no obvious reason for why a system is doing surprisingly better than those in the same situation [Pascale et al., 2010]). Therefore, the critical step of the bright spots framework is in digging into factors contributing to bright–dark classifications to generate ideas about underlying mechanisms that explain why an outlier is a bright–dark spot. Importantly, these mechanisms may relate to behaviors or circumstances that inherently cannot be modeled or easily observed at the outset (e.g., social factors, complex interactions, emergent properties) (Ostrom, 2007). By identifying factors contributing to unpredicted outcomes, this can generate ideas about potential intervention opportunities related to those mechanisms. For example, identifying bright spots in child growth in developing countries highlighted community structure and feeding practices as key factors for success; however, these drivers were not identified by a model but through additional qualitative observation of families with healthier kids than expected (Zeitlin, 1991). In aquatic ecology, a key example of this exploration of hidden mechanisms is in analyses of lake productivity. Log‐linear relationships between lake productivity (chlorophyll *a*) and lake total phosphorus have long been reported across multiple sites and scales of study (Dillon & Rigler, 1974; Havens & Nürnberg, 2004), leading to greater understanding of drivers and management of lake eutrophication. However, investigations into variability in the relationship between chlorophyll *a* and lake total phosphorus and factors associated with lakes with both higher and lower than expected production have spurred enormous insights (Quinlan et al., 2021). For example, by digging deeper into these sites, researchers hypothesized that food web interactions may be responsible for eutrophication, prompting whole‐lake experiments that revealed the importance of fish community structure and trophic relationships in regulating chlorophyll *a* relative to phosphorus inputs (Carpenter et al., 2001, 1985).

Often, the process of digging deeper into bright or dark spots is qualitative and can be based on anecdotal evidence (e.g., Gladwell, 2011). Qualitative methods can incorporate social dimensions that may be difficult to quantify, such as public perceptions or intrinsic values (Hunt et al., 2019; Stemmer et al., 2022). For instance, it may be important to incorporate public opinions on site performance into classifications because these perceptions can influence their support of potential actions regardless of actual site performance (Beard et al., 2003). Public perception of bright spots can also be used to identify bright spots for future analysis (e.g., leading into the iterative process in Figure 2). These qualitative approaches may be general (e.g., conversations with rightsholders and stakeholders to identify characteristics of spots) or specific (e.g., targeted observation of classified bright spots). Here, social science methods (e.g., ripple effect mapping [Chazdon et al., 2017]), structured decision frameworks (Runge & Walshe, 2014), or expert opinion‐informed Bayesian priors (Murray et al., 2009) may be useful in deciding what social and ecological attributes of sites denote a bright spot prior to further analyses.

The iterative application of the bright spots framework (Figure 2) can begin at multiple points in the process. Bright spots can be identified a priori based on qualitative or quantitative criteria, and then classification and context‐dependent factors can be used to explain what makes them bright. Alternatively, bright spots may be identified in initial modeling while controlling for nonactionable variables but are then explained by the addition of actionable factors and identification of hidden factors. Regardless, this approach enhances learning and operationalizes that knowledge for effective conservation and stewardship.

### Approach limitations

Defining expectations is challenging in the bright spots approach; all classifications for bright and dark spots are relative to a predicted outcome. Model predictions can vary significantly based on context, making it hard to establish universal benchmarks. Data availability and quality also affect outcomes; incomplete or biased datasets and the choice of predictors play crucial roles in classification accuracy and robustness (Elphick, 2008). Additionally, the bright spots approach, which compares expectations to observations, is not suitable for forecasting, as future observations are not available in the present. However, using the approach to better understand under‐ and overperformers may be valuable for improving projections. These constraints highlight the need for a nuanced understanding of the approach's limits and influencing factors.

## IMPLICATIONS FOR STEWARDSHIP

The process and classification of systems into bright spots, dark spots, hotspots, or coldspots we have outlined can enhance understanding of ecosystem processes and inform management. However, the implications for policy and decision‐making depend on context (Figure 4; Table 1). Each classification (i.e., bright, hot, dark, cold) and its combinations (i.e., bright–hot, bright–cold, dark–hot, dark–cold) can help identify influential drivers, both in and out of local control, and guide potential stewardship options. For example, bright spots that stay bright despite inclusion of additional predictors may be candidates for protection given the uncertain nature of their success (Gilby et al., 2023). Alternatively, bright spots that are explained and become hotspots could point to actions to increase success and may be candidates for either protection (e.g., biodiversity hotspot) or the expansion of opportunities (e.g., allowing more harvest in productive fisheries), depending on the identified mechanism. We highlighted examples of each classification as well as potential stewardship implications.

### Bright–hot

Distinct management options arise for bright–hot spots, which represent areas with large absolute and unpredictably high values of a response metric. When a status is robust (e.g., bright–hot), stewardship may prioritize protection. This approach has been adopted in biodiversity conservation to identify areas of greatest biodiversity to protect and prioritize for conservation (Figure 3). For example, Kovalenko et al. (2019) assessed patterns in functional and taxonomic fish biodiversity across coastal freshwater wetlands in the northern Laurentian Great Lakes to identify areas most likely to maximize conservation benefits based on biodiversity (response metric) relative to land use (expectation that diversity was low with intensive land use). For example, Kovalenko et al. (2019) analyzed patterns of functional and taxonomic fish biodiversity in coastal freshwater wetlands of the northern Laurentian Great Lakes. By comparing biodiversity (response metric) against expectations based on land‐use intensity (predictor), they identified outliers with exceptionally high taxonomic richness or functional diversity in heavily impacted watersheds. These bright–hot areas (high in biodiversity despite intensive land use) were interpreted as potentially resilient and thus prioritized for increased protection but could also be areas for expanded opportunities depending on the management objective (Figure 4)

### Bright–cold

Alternatively, bright–cold spots, which represent areas with low absolute values of a response metric that are still higher than predicted, may indicate where to focus management changes to improve low‐performing sites in priority areas. For example, to support declining coldwater fishes, Hansen et al. (2022) classified lakes based on oxythermal habitat availability (response metric) relative to watershed disturbance (expectation that habitat availability is lower with greater disturbance). They identified lakes with low availability of coldwater fishes’ habitat (cold) that nonetheless contained more habitat than predicted given their climatic and watershed conditions (bright) (Hansen et al., 2022). Although these lakes did not represent the best available habitat across the entire study region, they were highly resilient lakes that represented the best possible refuge for coldwater fishes in the southern extent of their range. In this case, simply classifying these systems as cold, but ignoring their brightness, could have resulted in managers overlooking their potential utility as resilient refugia for declining coldwater fishes in key regions. Coldwater fishes have declined extensively given climate change, so although habitat in these bright–cold lakes was limited, they represented the best remaining sites to prioritize for restoration through new actions (potentially experimental) (Walters & Hilborn, 1978) to support a species of concern (Hansen et al., 2022) (Figure 4).

### Dark–hot

Dark–hot spots, which represent systems with high absolute values of a response metric that are still unpredictably lower, offer an opportunity to alter current actions given these areas have potential for improved performance. For example, in response to amphibian declines due to fungal disease, Scheel et al. (2019) quantified areas of amphibian diversity (response metric) relative to disease prevalence (expectation that diversity is lower in areas with greater fungal disease). Through their analysis, they identified areas whose high amphibian diversity (hot) nonetheless failed to meet the very high levels of diversity predicted given their low disease prevalence (dark). These dark–hot locations suggest systems where new actions, potentially experimental, may improve understanding of factors beyond disease prevalence that are limiting diversity (Figure 4; Scheel et al., 2019).

### Dark–cold

Dark–cold spots represent areas with low absolute values of a response metric that are lower than predicted given their conditions. They offer opportunities to identify limiting factors and areas for improvement. Because they exist in an undesirable state relative to expectations, dark–cold spots offer opportunities for improvement in these unpredictably low‐performing systems. For example, urban green spaces (response metric) support environmental justice initiatives and community well‐being but are more prevalent in affluent communities (expectation that underserved, less affluent areas have less green space) (Wolch et al., 2014). Therefore, to identify areas that would most benefit from green‐space development, Wolch et al. (2014) proposed that areas lacking green space (cold), especially in less affluent areas (dark), are ripe for investment and should be prioritized through altered actions for green‐space development (Figure 4).

### Combined classifications

Bright–dark spot classification can inform natural resource conservation and stewardship by identifying influential drivers, including those under local control and those not easily identifiable through initial modeling. When placed in a landscape or multisystem context, classifications help balance priorities (e.g., social, biological, abiotic). For example, when classifying forests as bright based on ecological factors (e.g., tree density and diversity) and social factors (e.g., presence of culturally important species), the overlap of ecological and social bright spots can help guide management priorities to meet both ecological and social needs. Further, iteratively evaluating bright spots for various elements (e.g., species, habitats, social perception) in a single system can inform ecosystem‐based management. For instance, many inland fisheries are managed for single species despite broader food web interactions (Pikitch et al., 2004). Using the bright spots framework, if one were to classify spots for multiple species, bright–hot locations may be areas of high conservation priority. Combining multiscale, multispecies bright spots through this framework would help in the conservation and management of dynamic natural resources.

## CONCLUSION

Focusing on bright spots and examples of unexpected success offers new insights, social context, and more effective management possibilities. We provided unified definitions of bright spots, dark spots, hotspots, and coldspots and a flexible framework to classify systems. This bright spots framework involves a learning loop, involving setting expectations, classifying systems, and digging deeper into factors contributing to classifications. Regardless of classification, the iterative process we outlined can inform conservation and stewardship by enhancing understanding of ecological and social processes, identifying drivers of success, facilitating resource prioritization, and informing ecosystem‐based management.

The bright spots framework complements and extends several established conservation approaches, including refugia, resilience hubs, outlier analysis, structured decision‐making, the resist–accept–direct adaptation tool, and adaptive management. Like refugia and resilience hubs, bright spots draw attention to areas that support ecological integrity under pressure (Keppel et al., 2012). Refugia and resilience hubs often focus on inherent stability or resistance to change, and bright spots are systems that perform exceptionally well relative to expectations, often despite environmental stressors. This performance‐based perspective highlights not just persistence but also thriving and helps identify actionable drivers of success. The framework also aligns with outlier analysis by focusing on statistical deviations from predicted outcomes, providing a transparent and data‐driven method for highlighting exceptional cases (Russello et al., 2012). Additionally, bright spots can inform decision support approaches (e.g., structured decision‐making, the resist–accept–direct adaptation tool) by offering evidence‐based starting points for evaluating trade‐offs and targeting interventions (Hemming et al., 2022; Lynch et al., 2021). In contexts of uncertainty or ongoing change, bright spots may serve as learning opportunities in an adaptive management cycle, where identifying what works and why can guide iterative improvements in policy and practice (Walters & Hilborn, 1978). Together, these connections position the bright spots framework as a bridge between diagnostic insight and strategic action in conservation planning.

The future development of the bright spots approach could address several compelling opportunities. Inclusion of qualitative methods and data can enrich bright spot classifications by capturing social contexts and nuances that quantitative data alone may overlook. For example, combining multiple ways of knowing (e.g., traditional ecological knowledge and Western science) to inform expectations, classify systems, and explore factors contributing to success provides a broader view of dynamics and potential intervention options (Reid et al., 2020). There is also a growing need to apply statistical methods that account for spatial and temporal variability (Thorson & Kristensen, 2024). Recognizing these dimensions can better capture the dynamic nature of real‐world processes, leading to more accurate predictions and classifications. To further improve management prioritization, it is critical to establish a clear path from detection to action, given individual contexts. These advancements may lead to more comprehensive and dynamic bright spot classifications, offering deeper insights into complex systems and enhancing decision‐making.
